# On the Role of Theta-Driven Syllabic Parsing in Decoding Speech: Intelligibility of Speech with a Manipulated Modulation Spectrum

**DOI:** 10.3389/fpsyg.2012.00238

**Published:** 2012-07-16

**Authors:** Oded Ghitza

**Affiliations:** ^1^Hearing Research Center, Boston UniversityBoston, MA, USA

**Keywords:** speech perception, intelligibility, syllabic parsing, modulation spectrum, cascaded neuronal oscillations, theta band, hierarchical window structure, synchronization

## Abstract

Recent hypotheses on the potential role of neuronal oscillations in speech perception propose that speech is processed on multi-scale temporal analysis windows formed by a cascade of neuronal oscillators locked to the input pseudo-rhythm. In particular, Ghitza (2011) proposed that the oscillators are in the theta, beta, and gamma frequency bands with the theta oscillator the master, tracking the input syllabic rhythm and setting a time-varying, hierarchical window structure synchronized with the input. In the study described here the hypothesized role of theta was examined by measuring the intelligibility of speech with a manipulated modulation spectrum. Each critical-band signal was manipulated by controlling the degree of temporal envelope flatness. Intelligibility of speech with critical-band envelopes that are flat is poor; inserting extra information, restricted to the input syllabic rhythm, markedly improves intelligibility. It is concluded that flattening the critical-band envelopes prevents the theta oscillator from tracking the input rhythm, hence the disruption of the hierarchical window structure that controls the decoding process. Reinstating the input-rhythm information revives the tracking capability, hence restoring the synchronization between the window structure and the input, resulting in the extraction of additional information from the flat modulation spectrum.

## Introduction

There is a remarkable correspondence between the time span of phonetic, syllabic, and phrasal units, on the one hand, and the frequency range of the gamma, beta, theta, and delta neuronal oscillations, on the other. Phonetic features (mean duration of 25 ms) are associated with gamma (>40 Hz) and beta (15–35 Hz) oscillations, syllables and words (mean duration of 250 ms) with theta oscillations (4–8 Hz), and sequences of syllables and words embedded within a prosodic phrase (500–2000 ms) with delta oscillations (<3 Hz). This correspondence inspired recent hypotheses on the potential role of neuronal oscillations in speech perception (e.g., Poeppel, 2003; Ahissar and Ahissar, 2005; Ghitza and Greenberg, 2009; Ghitza, 2011; Giraud and Poeppel, 2012; Zion-Golumbic et al., 2012). In particular, in an attempt to account for counterintuitive behavioral findings on the intelligibility of time-compressed speech as a function of “repackaging” rate (Ghitza and Greenberg, 2009), Ghitza (2011) proposed a computation principle where the speech decoding process is controlled by a time-varying hierarchical window structure *locked* to the input pseudo-rhythm. The key property that enabled an explanation of the behavioral data was the capability of the window structure to stay *synchronized* with the input; performance is high so long as the oscillators are locked to the input rhythm (and within their intrinsic frequency range), and it drops once the oscillators are out of lock (e.g., hit their boundaries).

**Figure 1:**
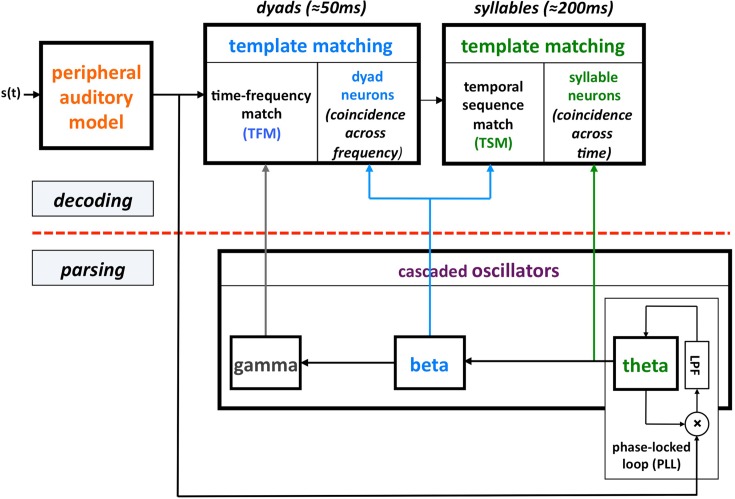
**A block diagram of the Tempo model**. It comprises lower and upper paths that process the sensory stream generated by a model of the auditory periphery. The lower path extracts **parsing** information, which controls the **decoding** process performed in the upper path. The parsing is expressed in the form of a *time-varying hierarchical window structure*, realized as an array of cascaded oscillators locked to the input syllabic rhythm; the frequencies and relative phases of the oscillations determine the temporal windows (location and duration) that control the decoding process. See text for details.

This computation principle was realized by the phenomenological model shown in Figure 1, termed *Tempo*. In this model the sensory stream is processed, simultaneously, by a *parsing* path and a *decoding* path, which correspond to the lower and upper parts of Figure 1. Conventional models of speech perception assume a strict decoding of the acoustic signal. The decoding path of Tempo conforms to this notion; the decoding process links chunks of sensory input with different durations with stored linguistic memory patterns. The additional, parsing path determines a temporal window structure (location and duration) that controls the decoding process. The windows are defined as the cycle-duration of oscillations in the theta, beta, and gamma frequency bands, all cascaded and locked to the pseudo-rhythmic speech input. The theta oscillator, capable of tracking the input syllabic rhythm, is the *master*; the other oscillators entrain to theta thus forming a hierarchy of analysis windows synchronized with the input. The theta oscillator provides *syllabic* parsing; assuming a perfect tracking, a theta cycle is aligned with a syllable that is often a [Vowel]–[Consonant-cluster]–[Vowel]. (This is so because the prominent energy peaks across the auditory channels, which presumably feed the theta tracker, are associated with vowels.) The *phonetic* temporal windows are determined by the cycles of the beta (entrained to theta). The rationale behind proposing the theta as the master oscillator is the robust presence of energy fluctuations in the 3- to 9-Hz range in the temporal auditory response to speech acoustics. Note that the term “parsing” as employed here does not refer to the exhaustive division of the incoming speech signal into candidate constituents, or even the inference of candidate constituents from the cues in the speech signal, but rather to the function of setting a time-varying hierarchical window structure synchronized to the speech input. (See Ghitza, 2011 for a detailed description of Tempo.)

The purpose of the study reported here was to provide evidence for the hypothesized role of theta in syllabic parsing. This was achieved by measuring the intelligibility of speech with manipulated modulation spectrum. Two related observations, reported by Chait et al. (2005) and Saoud et al. (2012), are noteworthy. In both these studies critical-band envelopes were decomposed into low (0–4 Hz) and high (22–40 Hz) components, each carries syllabic or phonetic information, respectively. Subjects heard naturally spoken sentences (in Chait et al., 2005), or words in isolation (in Saoud et al., 2012), and were instructed to type the words heard. Importantly, correct scores of 18/41% (Chait et al., 2005), and 20/95% (Saoud et al., 2012), were reported for a high/low component, respectively, suggesting a dominant role of the low component (associated with the delta and theta rhythms). In the context of our study, a question arises whether this dominance should be attributed to the syllabic parsing function of the theta, to the improved performance of the decoding path (when presented with richer spectro-temporal information), or to both.

To sharpen the association between the error patterns and the underlying mechanism for their cause we propose different kind of manipulations. Two classes of stimuli are envisioned: Class-I stimuli, with acoustic-phonetic information sufficient for the function of the decoding path, but without information about the input syllabic rhythm; and Class-II stimuli, with extra information, restricted to the input syllabic rhythm alone, re-inserted into the modulation spectrum of Class-I stimuli. Intelligibility of Class-I stimuli is expected to be poor: the absence of input-rhythm information should prevent the theta oscillator from tracking the input rhythm, hence disrupting the hierarchical window structure that controls the decoding process. Improved intelligibility of Class-II stimuli could be attributed to the reinstatement of theta parsing capability, hence the recovery of synchronization between the window structure and the input, resulting in the extraction of additional information from the flat modulation spectrum.

## Materials and Methods

### Stimulus preparation

The study comprises two experiments distinguished by the signal processing strategy used for the manipulation of the modulation spectrum.

#### Experiment I: peak position coding (PPC) of critical-band envelopes

**Figure 2:**
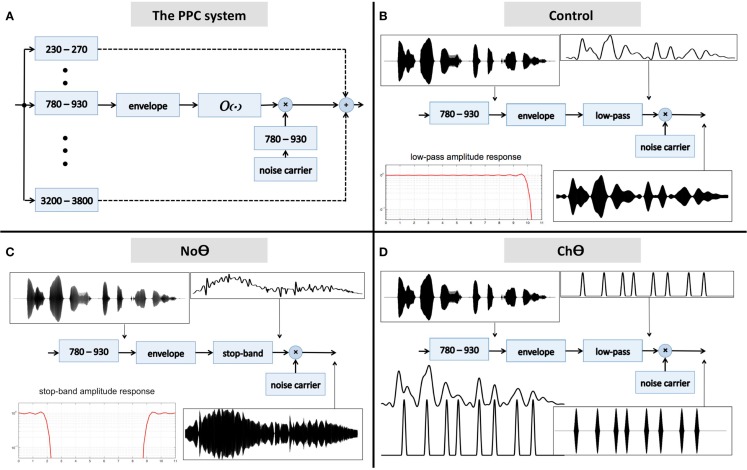
**(A)** A block diagram of the peak position coding (PPC) system used in Experiment I. The signal at the system output is the linear sum of all critical-band channels. The core experimental conditions are defined by the operator O. **(B)** The **Control** condition. Operator O_1_ is a low-pass filter, with a cutoff frequency of 10 Hz. **(C)** The **No-Theta (NoΘ)** condition. Operator O_2_ is a stop-band filter with a 2- to 9-Hz frequency gap. **(D)** The **Channel-Theta (ChΘ)** condition. Operator O_3_ comprises a linear low-pass filter (cutoff frequency of 10 Hz) followed by a peak picking operation. The pulse train represents, in a minimalistic form, the input-rhythm information at this frequency channel. See text for details.

The block diagram of the system is shown in Figure 2A. The input waveform is filtered by a bank of critical-band filters, which span the 230- to 3800-Hz frequency range. Each critical-band channel is processed in the same manner: the envelope of the filter’s band-limited output is derived first (e.g., the Hilbert envelope), followed by an operator O. The output of the operator modulates a narrow-band noise carrier centered at the mid frequency range of the critical-band, with a bandwidth equal to critical-band and with instantaneous phase which minimizes noise-envelope fluctuations. (The goal is to have a carrier with a fine structure that is independent of the fine structure of the band-limited signal, to prevent a regeneration of the original critical-band envelope at the listener’s cochlear output; Ghitza, 2001.) The signal at the system output is the linear sum of all critical-band channels.

**Figure 3:**
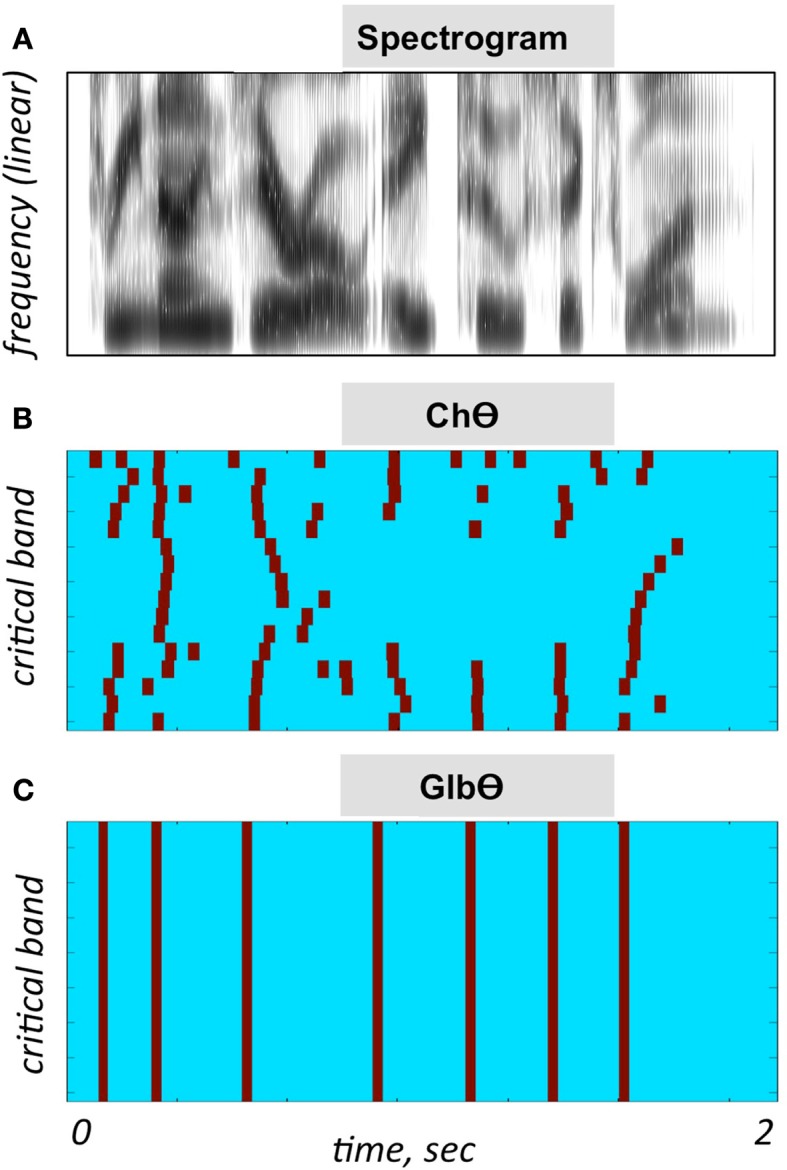
**Illustration of the PPC strategies used in Experiment I**. **(A)** A spectrogram of the stimulus used for **(B,C)**. Abscissa is time and the ordinate is frequency, in linear scale. **(B)** A ChΘ strategy. Pulses are placed at the peak location of the filtered critical-band envelopes. Abscissa is time and the ordinate is critical-band (i.e., frequency in a critical-band scale). Note the curvature in the pulse contours, indicating a residue of spectro-temporal information. **(C)** A GlbΘ strategy. A single pulse train is generated, same for all channels, with pulses at the mid-vowel locations obtained by hand-segmenting the full-band signal. Note that the location of the mid-vowel is loose, within a time interval in the order of a few pitch cycles.

**Figure 4:**
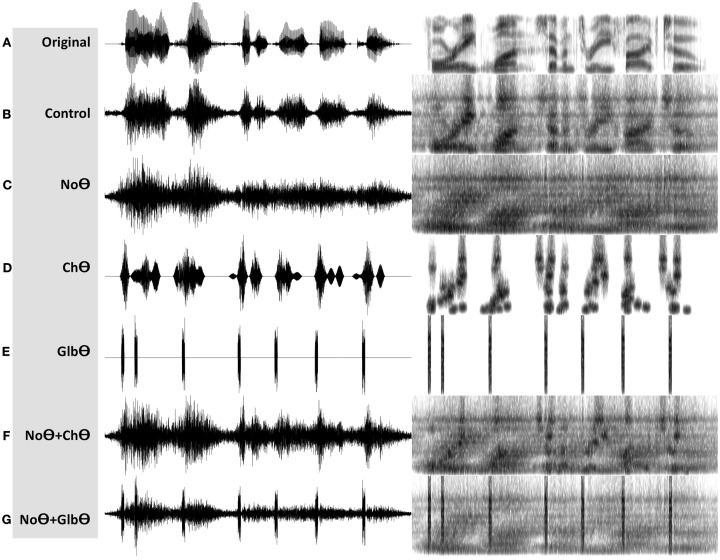
**Waveforms (left hand side) and spectrograms (right hand side) generated by the PPC system (Figure 2A)**. The stimuli are 4 kHz wide. The frequency range of the spectrograms is 0–5 kHz. MP3 files are available for listening as Supplementary Materials. **(A)** The unprocessed waveform. **(B)** A **Control** stimulus, generated by the system shown in Figure 2B. **(C)** A **NoΘ** (No-Theta) stimulus, generated by the system shown in Figure 2C. **(D)** A **ChΘ** (Channel-Theta) stimulus, generated by the system shown in Figure 2D. **(E)** A **GlbΘ** (Global-Theta) stimulus, generated by replacing ChΘ pulses with a single pulse train – same for all channels – located at mid-vowel locations obtained by hand segmentation of the full band signal (Figure 3C). **(F)** A **NoΘ + ChΘ** stimulus – a linear sum of a NoΘ stimulus plus the corresponding ChΘ stimulus. **(G)** A **NoΘ + GlbΘ** stimulus – a linear sum of a NoΘ stimulus plus the corresponding GlbΘ stimulus.

Five experimental conditions were tested:

1. A **Control** condition, generated by operator O_1_ (Figure 2B). This operator is a low-pass filter, with a cutoff frequency of 10 Hz and a frequency response shown in the lower-left insert. The band-limited signal, the filtered envelope and the modulated noise carrier are shown at the upper-left, upper-right, and lower-right inserts, respectively. The filtered envelope contains acoustic-phonetic information sufficient for speech comprehension (e.g., Drullman et al., 1994). As such, stimuli in this condition are expected to be highly intelligible. Figure 4B shows the waveform (left) and the spectrogram (right) of a Control stimulus. An MP3 file of the depicted stimulus is available for listening as Supplementary Materials.
2. A **No-Theta (NoΘ)** condition, generated by operator O_2_ (Figure 2C). This operator is the stop-band filter shown in the lower-left insert, with a 2- to 9-Hz frequency gap. The band-limited signal, the filtered envelope, and the modulated noise carrier are shown at the upper-left, upper-right, and lower-right inserts, respectively. As reflected in the upper-right insert the filtered envelope does not contain syllable-rate fluctuations. As such, the nullification of the 2- to 9-Hz modulation-frequency band is expected to considerably reduce intelligibility. See example in Figure 4C.
3. A **Channel-Theta (ChΘ)** condition. Operators O_1_ and O_2_ are linear operators. Operator O_3_, depicted in Figure 2D, is non-linear in nature, comprises a linear low-pass filter (cutoff frequency of 10 Hz) followed by a peak picking operation. The lower-left insert illustrates the operator; the upper trace shows the filtered envelope and the lower trace – a sequence of pulses, Hamming-window shaped and identical in amplitude, located at the peaks of the filtered envelope. This operator is termed *Peak Position Coding* (or *PPC*), and the pulse train represents the input rhythm in a minimalistic form. The band-limited signal, the PPC envelope, and the modulated noise carrier are shown at the upper-left, upper-right, and lower-right inserts, respectively. Figure 3B illustrates the sparseness of the pulse distribution in the time-frequency plane for a 2-s long speech input, whose spectrogram is shown in Figure 3A. The stimuli in this condition are expected to be barely intelligible. See example in Figure 4D.
4. A **NoΘ + ChΘ** condition. This stimulus is a linear sum of a NoΘ stimulus plus the corresponding ChΘ stimulus. See example in Figure 4F.
5. A **NoΘ + GlbΘ** condition. Although much of the acoustic-phonetic information in ChΘ signals was removed, a residue of spectro-temporal information is still present (indicated by the curvature in the contours of Figure 3B). To further reduce the acoustic-phonetic information we replaced the channel-theta (ChΘ) with a **Global-Theta (GlbΘ)** version: instead of placing pulses at the peak location of the filtered envelope per critical-band, a single pulse train is generated – same for all channels – with pulses at mid-vowel locations obtained by hand segmentation of the full-band signal (Figure 3C). Note that the location of the mid-vowel is loose, within a time interval in the order of a few pitch cycles. See example of GlbΘ and NoΘ + GlbΘ signals in Figures 4E,G respectively.

MP3 files are available for listening as Supplementary Materials.

#### Experiment II: infinite-clipping (InfC) of critical-band signals

**Figure 5:**
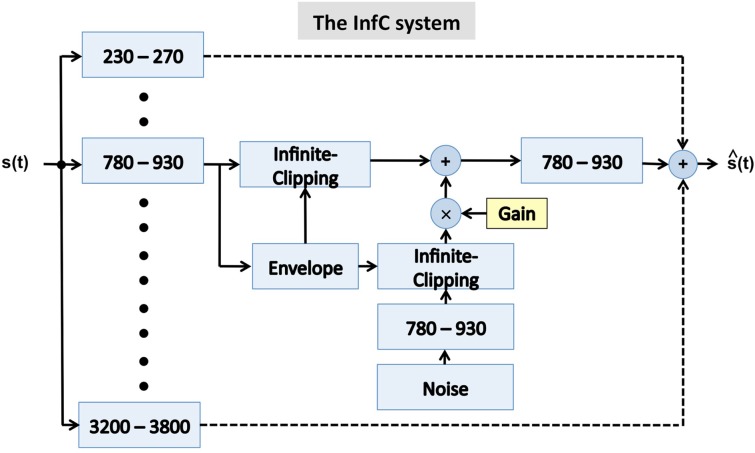
**A block diagram of the infinite-clipping (InfC) system used in Experiment II**. The signal at the system output is the linear sum of all critical-band channels. The system is a generalization of a design used by Licklider and Pollack (1948), where an infinite-clipping operator was applied to the full-band signal. Here, the infinite-clipping operation is applied to each critical-band output (prior to summation). The parameter Gain determines the degree of critical-band envelope flatness. See text for details.

In a pursuit of better separation between the role of syllabic parsing and the role of decoding, we used yet another speech processing strategy shown in Figure 5. In Experiment I, NoΘ stimuli were contrasted with NoΘ + ChΘ stimuli. Here, the intelligibility of stimuli with varying degree of critical-band envelope flatness was measured. The system is a variation of a design used by Licklider and Pollack (1948); there, the full-band speech was passed through a 1-bit hard limiter (i.e., an infinite-clipping operation), resulting in highly intelligible stimuli. Here we generalize this principle, with the infinite-clipping operation applied to the critical-band signals (prior to summation).

**Figure 6:**
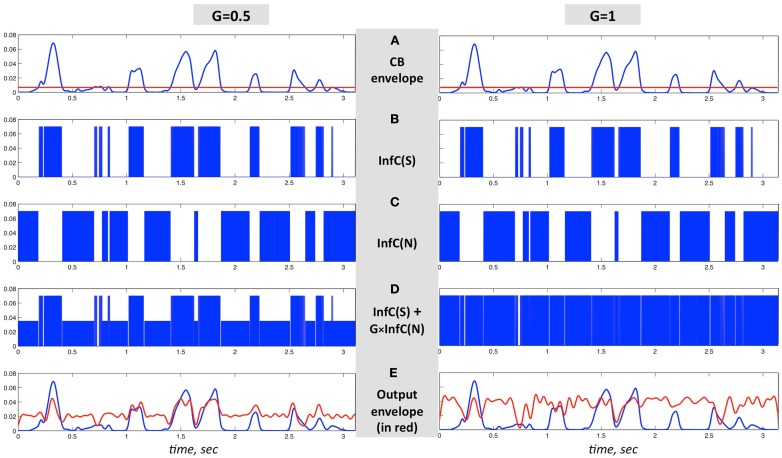
**An illustration of the signal flow in one critical-band channel of the system in Figure 5 for *G* = 1 (right column) and *G* = 0.5 (left column)**. **(A–C)** are same in both columns. **(A)** The envelope of a band-limited signal, low-pass filtered to 10 Hz. **(B)** InfC(S) is the binary output of an infinite-clipping operator, operating on *non-zero signal intervals* – intervals where the envelope is above a prescribed fixed threshold [**(A)**, red horizontal line]. **(C)** InfC(N) is the binary output of an infinite-clipping operator operating on band-limited noise, only inside the gaps in-between non-zero signal intervals. **(D)** The sum of **(B,C)**, with Gain values *G* = 0.5 (left) and *G* = 1 (right). The channel output is the signal of **(D)**, band-pass filtered by a *postfilter* identical to the channel critical-band filter. **(E)** The envelope of the channel output, in red, overlaid on top of the blue curve from **(A)**.

The critical-band filters span the 230- to 3800-Hz frequency range. All critical-band channels are processed in the same manner, as illustrated in Figure 6. Figures 6A–C are same in both columns; Figure 6A shows the envelope of the band-limited signal, low-pass filtered to 10 Hz. Figure 6B shows the binary output of an infinite-clipping operator operating on the band-limited signal, but only at intervals with envelope above a prescribed fixed threshold (Figure 6A, red horizontal line); those intervals are termed *non-zero signal intervals*. Figure 6C shows the binary output of an infinite-clipping operator operating on noise, only inside the gaps between non-zero signal intervals. The noise is centered at the mid frequency range of the critical-band, with a bandwidth equal to the critical-band. Figure 6D shows the sum of Figures 6B,C, with Gain-parameter values *G* = 0.5 (left) and *G* = 1 (right). The channel output is the signal of Figure 6D band-pass filtered by a *postfilter*, identical to the channel critical-band filter. The envelope of the channel output (Figure 6E, red curve) is overlaid on top of the blue curve from Figure 6A.

A few properties of the system are worth noting here:

1. It is known that the fine structure (e.g., the Hilbert phase) of a band-limited signal contains information on its temporal envelope (e.g., Voelcker, 1966). It is also known that when a signal – generated by flattening the temporal envelope of a band-limited signal while keeping the fine structure untouched – is passed through a band-pass filter with a center frequency equal to the center frequency of the band-limited signal, the rich temporal envelope of the original signal is regenerated, to a large extent, at the output (Ghitza, 2001). In our context, the binary output of the non-zero signal intervals maintains the fine structure of the signal itself. Therefore, when passed through the postfilter, envelope information is surfaced at the output. If the postfilter is viewed as a listener’s cochlear channel at the corresponding frequency, then the postfilter output (red curve of Figure 6E) reflects the envelope at the listener’s cochlear output. (Note that, strictly speaking, the critical-band filters are auditory filters derived from psychophysical data rather than cochlear filters derived from physiological measurements. In the context of our study, however, this difference is not relevant.)
2. The role of the postfilter is to limit the bandwidth of the binary signal in Figure 6D – a wideband signal in nature – to the critical-band frequency band. Without the inclusion of a postfilter, the summation of channels will result in the spill of noise from one channel onto neighboring channels, adding noise to the non-zero signal intervals of the neighboring channels. (This is so because the locations of the non-zero signal intervals are channel specific and not necessarily time aligned). The postfilter prevents such interference.
3. An important property of the system is that inside the non-zero signal intervals the critical-band binary output, and thus the envelope at the postfilter output (due to property 1), are the same for all *G* (this is so because binary noise, calibrated by *G*, is added only in gaps in-between the non-zero signal intervals). Therefore, the acoustic-phonetic information *in the cochlear response* to the system output signal (i.e., after summation) is also independent of *G* (due to property 2).
4. The degree of envelope flatness is controlled by the Gain parameter. For example, for *G* = 1 the envelope of the signal of Figure 6D is 100% flat; for *G* = 0.5 the envelope exhibits input syllabic rhythm. As stated in property 3, the spectro-temporal information in the cochlear response to non-zero signal intervals is independent of gain. Therefore, any difference in intelligibility between two stimuli with different gain values (say *G* = 1 vs. *G* = 0.5) could be attributed only to the recovery of syllabic parsing.
5. For *G* = 1, the temporal envelope should convey zero information about the input syllabic rhythm. Even though sharp envelope fluctuations may occur at the boundaries of the non-zero signal intervals, the temporal envelope at the output of the postfilter (red curve of Figure 6E) conveys little information on the input rhythm.

In Experiment II four conditions were tested, with varying degree of flatness: ***G*** = **0** (zero flatness), ***G*** = **1** (100% flatness), and two in-between conditions, ***G*** = **0.5** and ***G*** = **0.8**. The intelligibility of *G* = 1 stimuli is expected to be poor; significant improvement is expected for *G* = 0.5 and = 0.8 stimuli.

**Figure 7:**
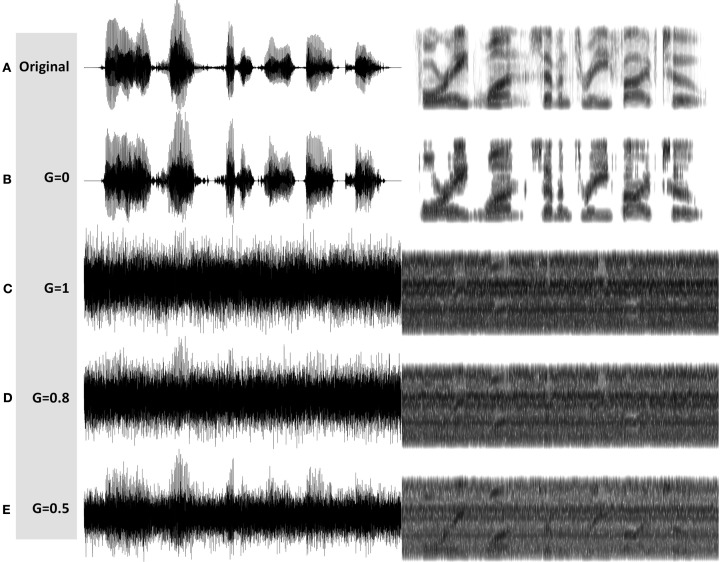
**Waveforms (left hand side) and spectrograms (right hand side) generated by the InfC system (Figure 5)**. The stimuli are 4 kHz wide. The frequency range of the spectrograms is 0–5 kHz. MP3 files are available for listening as Supplementary Materials. Shown are the unprocessed waveform and four processed stimuli, with varying degree of critical-band envelope flatness controlled by the parameter *G*: **(A)** The unprocessed waveform. **(B)** A ***G* = 0** stimulus, with zero flatness. **(C)** A ***G* = 1** stimulus, with 100% flatness. **(D)** A ***G* = 0.8** stimulus, and **(E)** a ***G* = 0.5** stimulus, with in between degree of flatness.

See examples of *G* = 0, = 1, = 0.8, and = 0.5 stimuli in Figures 7B–E, respectively. MP3 files of the depicted stimuli are available for listening as Supplementary Materials.

### Subjects

All listening subjects were young adults (college students) educated in the U.S. with normal hearing. Ten subjects participated in Experiment I; different set of subjects, 10 in number, participated in Experiment II. Although the number of listeners is smaller than typical for this type of study, their results (as described in Section “Results”) are consistent with each other.

### Corpus

The experimental corpus comprised 100 digit strings spoken fluently by a male speaker. Each string is a seven-digit sequence and is approximately 2 s long. It is uttered as a phone number in an American accent, i.e., a cluster of three digits followed by a cluster of four digits (for example: “two six two, seven O one eight”). It is a low perplexity corpus (a vocabulary of 11 words, 0–9, and O) but without contextual information. For each signal-manipulation condition, 80 stimuli (out of 100) were chosen at random and concatenated in a sequence: [alert tone] [digit string] [5-s long silence gap] [alert tone] …

### Paradigm

Subjects performed the experiment in an isolated office environment (no other occupants) using headphones. There were two listening sessions for each signal-manipulation condition, *Training* and *Testing*. A training set contained 10 digit strings and a testing set contained 80 digit strings (approximately 12 min to complete). Training preceded testing; in the training phase, subjects had to perform above a prescribed threshold before proceeding to the testing phase. Subjects were instructed to listen to a digit string *once* and, during the 5-s long gap following the stimulus, to type into an electronic file the *last four digits* heard, in the order presented (always four digits, even those that she/he was uncertain about). The rational behind choosing the last four digits as target (as opposed to choosing the entire seven-digit string) was twofolded. First, it was an attempt to provide the opportunity for the presumed (cortical) theta oscillator to entrain to the input-rhythm prior to the occurrence of the target words (recall the inherent rhythm in the stimuli, being a seven-digit phone number uttered in an American accent). Second, it aimed at reducing the bias of memory load on the error patterns.

The human-subjects protocol for this study was approved by the Institutional Review Board of Boston University.

## Results

### Analysis procedure

Data is presented in terms of *error rate* and *normalized error rate*. Error rate was calculated by using two distinct error metrics: (i) *digit-error rate*, defined as the number of digits erroneously registered divided by the total number of digits (i.e., 80 × 4 = 320 digits), in percent, and (ii) *string-error rate*, defined as the number of four-digit strings that – as a whole – were erroneously registered, divided by the total number of strings (i.e., 80 strings), in percent. Error rates were calculated per metric, per condition, per subject. The bar charts show the mean and the standard deviation across subjects. (The standard deviation here is the square root of the unbiased estimator of the variance.)

In order to exclude inter-subject variability, *normalized error rates* were calculated by normalizing the raw scores per metric, per subject, relative to the flat envelope condition (NoΘ in Experiment I; *G* = 1 in Experiment II). Normalized errors show the *pattern* of change in performance re flat envelope condition.

The conclusiveness of the experimental data was tested via analysis of variance (ANOVA; see Section “Statistical Analysis”).

### Experiment I

**Figure 8:**
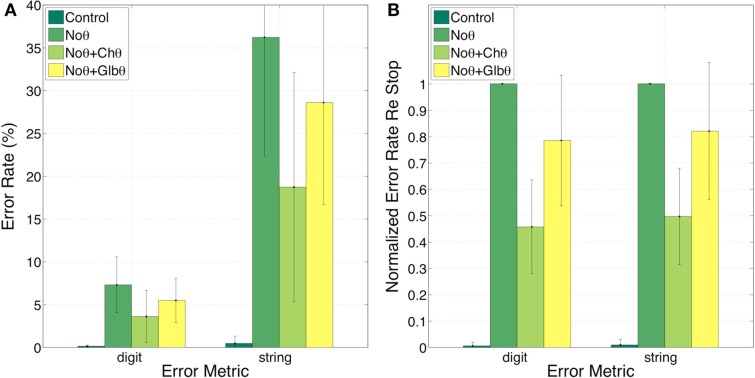
**Experiment I**. Error rates **(A)** and Normalized error rates **(B)** for conditions Control, NoΘ, NoΘ + ChΘ and NoΘ + GlbΘ. Two error metrics, digit-, and string-error, are used (defined in Section “Analysis Procedure”). Adding ChΘ or GlbΘ stimulus to the corresponding NoΘ stimulus improves intelligibility. Normalized error patterns indicate strong degree of consistency across subjects for the NoΘ vs. NoΘ + ChΘ contrast. A drop in the degree of consistency is noticed for the NoΘ vs. NoΘ + Glb**Θ** contrast. These observations are quantified in the analysis of variance (ANOVA) presented in Section “Statistical Analysis” (see Table 1).

Figure 8 shows the error rates (Figure 8A) and the normalized error rates (Figure 8B) for the Control, NoΘ, NoΘ + ChΘ, and NoΘ + GlbΘ conditions. As expected, the intelligibility of the Control stimuli is near perfect [see item 1 of Section “Experiment I: peak position coding (PPC) of critical-band envelopes”]. Comparing NoΘ to Control, errors jump from 0 to 7% (digit) and from 0 to 36% (string). For the task in hand (a low perplexity corpus and a limited memory load) such volume of errors is high. Re-inserting the ChΘ signal improves performance. Comparing NoΘ to NoΘ + ChΘ, errors drop from 7 to 4 (digit), and from 36 to 19 (string). Normalized NoΘ + ChΘ errors (re NoΘ) are 0.45 (digit) and 0.50 (string). Is the cause for this improvement the reactivation of the parsing path, the improved performance of the decoding path (due to extra spectro-temporal information still exists in ChΘ), or both?

**Table 1:** **A *post hoc* Tukey/Kramer test, performed independently on each of the data sets behind the figures listed in the left column, and per error-metric**.

	Error-metric	
	Digit	String
Figure 8A	NoΘ, NoΘ + ChΘ and NoΘ + GlbΘ are significantly different from Control	Same
	NoΘ + ChΘ is significantly different from NoΘ	
	NoΘ + GlbΘ has no significant difference from NoΘ	
	NoΘ + GlbΘ has no significant difference from NoΘ + ChΘ	
Figure 8B	Control, NoΘ + ChΘ and NoΘ + GlbΘ are significantly different from each other	Same
Figure 9	Control, NoΘ and ChΘ are significantly different from each other	Same
Figure 10A	*G* = 0, = 0.5, = 0.8 are significantly different from *G* = 1	Same
	No significant difference among *G* = 0, = 0.5, = 0.8	
Figure 10B	*G* = 0, = 0.5 are significantly different from *G* = 0.8	Same
	No significant difference among *G* = 0, = 0.5	

**Figure 9:**
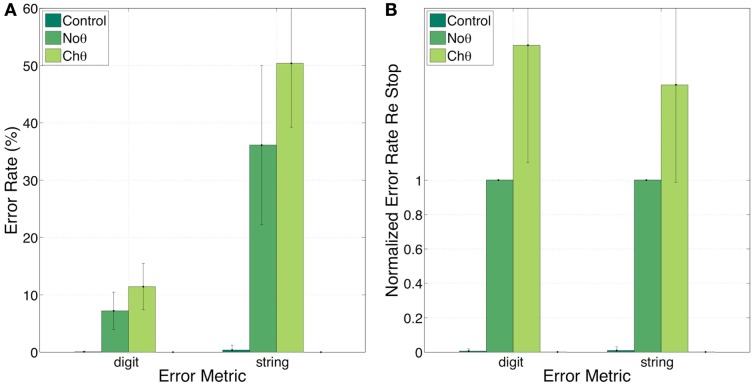
**Experiment I**. error rates **(A)** and normalized error rates **(B)** for conditions control, NoΘ, and ChΘ (alone). Intelligibility of ChΘ stimuli is worse than that of NoΘ stimuli, yet performance is better than chance, suggesting the existence of acoustic-phonetic information residue in the ChΘ stimuli, and indicating an imperfect disassociation between the roles of parsing and decoding.

Figure 9 provides a partial answer to this question. It shows errors, in percent, for the Control, NoΘ, and ChΘ (alone) conditions, with 0, 7, and 12 (digit) and 0, 36, and 50 (string), respectively. Intelligibility of ChΘ stimuli is worse than that of NoΘ stimuli, yet performance is better than chance, suggesting the existence of acoustic-phonetic information residue in the ChΘ stimuli, and indicating an imperfect disassociation between the roles of parsing and decoding.

In contrast, GlbΘ stimuli carry minimal (if any) acoustic-phonetic information. Comparing NoΘ to NoΘ + GlbΘ (Figure 8), errors drop from 7 to 5 (digit), and from 36 to 29 (digit); the corresponding normalized errors (re NoΘ) are 0.78 (digit) and 0.82 (string). As discussed in item 5 of Section “Experiment I: peak position coding (PPC) of critical-band envelopes,” this improvement is exclusively due to the recovered function of syllabic parsing.

Three observations are noteworthy. First, note the similarity in normalized error patterns between the digit- and string-error metrics. Second, the standard deviation bars in the normalized error patterns indicate strong degree of consistency across subjects for the NoΘ vs. NoΘ + ChΘ contrast. And third, a drop in the degree of consistency is noticed for the NoΘ vs. NoΘ + GlbΘ contrast. These observations will be quantified in the ANOVA presented in Section “Statistical Analysis.”

### Experiment II

**Figure 10:**
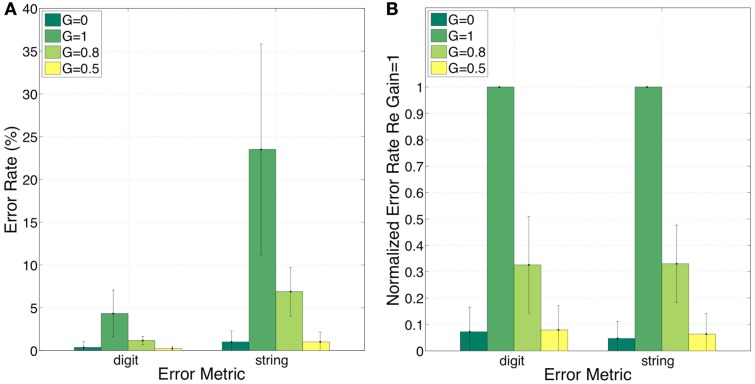
**Experiment II**. Error rates **(A)** and normalized error rates **(B)** for conditions *G* = 0, = 1, = 0.8, and = 0.5. Two error metrics, digit-, and string-error, are used (defined in Section “Analysis Procedure”). Reducing gain (i.e., reinstating critical-band envelope fluctuations) markedly improves intelligibility. Improvement is due exclusively to the recovered function of syllabic parsing (see property 4 of Section “Experiment II: infinite-clipping (InfC) of critical-band signals”). Note the consistency of normalized error patterns across subjects. These observations are quantified in the analysis of variance (ANOVA) presented in Section “Statistical Analysis” (see Table 1).

Figure 10 shows the error rates (Figure 10A) and the normalized error rates (Figure 10B) for the conditions *G* = 0, = 1, = 0.8, and = 0.5. As expected, the intelligibility of the *G* = 0 stimuli is near perfect. Comparing *G* = 1 to *G* = 0, errors jump from 0 to 4% (digit), and from 1 to 24% (string). For the task in hand (a low perplexity corpus and a limited memory load) such volume of error is high. Reducing the gain (i.e., reinstating critical-band envelope fluctuations) markedly improves intelligibility; comparing *G* = 1 to *G* = 0.8 (or *G* = 0.5), errors drop from 4 to 1 (or 0)% (digit), and from 24 to 7 (or 1)% (string). Normalized errors (re *G* = 1) are 0.07, 0.33, and 0.08 (digit), and 0.05, 0.33, and 0.06 (string), for *G* = 0, = 0.8, and = 0.5, respectively. As discussed in property 4 of Section “Experiment II: infinite-clipping (InfC) of critical-band signals,” this improvement is exclusively due to the recovered function of syllabic parsing. Note the consistency of normalized error patterns across subjects, and the similarity in normalized error patterns between the digit- and string-error metrics. This observation is quantified in the ANOVA presented next.

### Statistical analysis

An ANOVA was used to quantify the statistical significance of the data illustrated in Figures 8–10. Three factors were used, *system-type* (PPC vs. InfC), *condition* ([Control/*G* = 0] vs. [NoΘ/*G* = 1] vs. [NoΘ + ChΘ/*G* = 0.5] vs. [NoΘ + GlbΘ/*G* = 0.8]) and *error-metric* (digit vs. string). Note that the variables in the condition factor, in particular [NoΘ + ChΘ/*G* = 0.5] and [NoΘ + GlbΘ/*G* = 0.8], were lumped somewhat arbitrarily.

Mauchly’s test for sphericity revealed that assumptions of sphericity were not violated. The three-way ANOVA revealed that there is a significant main effect of each of the factors: (i) system-type (*F* = 49.08, *p* < 0.0001), (ii) condition (*F* = 46.19, *p* < 0.0001), and (iii) error-metric (*F* = 118.19, *p* < 0.0001). It also revealed that there is a significant interaction between each of the pairs: (i) system-type and condition (*F* = 7.05, *p* = 0.0002), (ii) system-type and metric (*F* = 22.36, *p* < 0.0001), and (iii) condition and metric (*F* = 21.27, *p* < 0.0001). A *post hoc* Tukey/Kramer test, performed independently on each of the data sets and per error-metric, revealed the relationships summarized in Table 1.

The ANOVA reinforces the trends observed in Sections “Experiment I” and “Experiment II,” i.e., that the intelligibility of stimuli with flat critical-band envelopes is poor, and that the addition of extra information, restricted to the input syllabic rhythm alone, significantly improves intelligibility.

## Discussion

In the larger context, this study should be viewed as one more step toward the validation of a model of speech perception (Tempo) with a brain-rhythms function at the core. The model hypothesizes that the speech decoding process is controlled by a time-varying hierarchical window structure locked to the input pseudo-rhythm; and that the neuronal realization of the window structure is in the form of a cascade of oscillators, with theta as the master. Do behavioral data exist in support of this hypothesis? We are aware of one set of such data, Ghitza and Greenberg (2009), upon which Tempo was developed.

The present study focuses on providing psychophysical evidence for the presumed role of theta. The methodology exploited here was to compare the intelligibility of two distinct classes of stimuli which, ideally, would have the following properties. Class-I stimuli would carry acoustic-phonetic information sufficient for the function of the decoding path but, at the same time, would have zero information about the input rhythm, to neutralize the parsing path; members in this class are referred to as stimuli with “flat” critical-band envelopes. Class-II stimuli, termed stimuli with “fluctuating” critical-band envelopes, are the sum of two signals: the signal from the first class plus a signal that would carry information restricted to the input rhythm alone (i.e., without any acoustic-phonetic content), aiming at reactivating the parsing path. Two signal processing systems were used to generate these stimuli, the PPC system [see Section “Experiment I: peak position coding (PPC) of critical-band envelopes”], and the InfC system [see Section “Experiment II: infinite-clipping (InfC) of critical-band signals”]. The psychophysical task – to listen to a seven-digit sentence (a phone telephone number) and to register the last four-digit sequence – is one with low corpus perplexity and with limited memory load.

### Full-band vs. critical-bands

When considering the possible role of the temporal envelope of speech in speech perception, the term “envelope” is often refers to the envelope of the waveform itself, i.e., of the full-band signal (e.g., Ahissar et al., 2001; Giraud and Poeppel, 2012; Zion-Golumbic et al., 2012). In this study, the signal processing manipulations are performed on the critical-band outputs. The sole acoustic input available to the brain is, by necessity, the information conveyed by the auditory nerve. In the context of studying cortical mechanisms for speech perception, a more insightful approach would be to consider the information available to the brain at the cochlear output level. To exemplify the benefit of such approach consider the difference between the time-frequency representations caricatured in Figures 3B,C. Figure 3B depicts a minimalistic representation of the input rhythm at the cochlear output while Figure 3C depicts the input rhythm at the waveform level. If the hypothesized role of theta (i.e., tracking the input syllabic rhythm) is correct, does the neuronal mechanism that tracks the input rhythm (e.g., a neuronal PLL circuitry; Ahissar et al., 1997) exploits the information embedded in the curvature of the pulse contours of Figure 3B?

### The PPC system vs. the InfC system

Chronologically, the PPC system was developed first. Conceptually, it aimed at improving the disassociation between the roles of parsing and decoding compared to the isolation provided by the strategy used by Chait et al. (2005) and Saoud et al. (2012). The InfC system emerged later, as a solution to shortcomings of the PPC system.

With the PPC system, Class-I stimuli are the NoΘ stimuli, composed of critical-band envelopes with null energy inside the 2- to 9-Hz modulation-frequency band. As such, these signals are stripped not only from input-rhythm information but also from a significant amount of acoustic-phonetic information (e.g., Houtgast and Steeneken, 1985; Drullman et al., 1994). One consequence of this deficiency is that the PPC strategy cannot be generalized to tasks with higher perplexity (see Section “Perplexity of Corpus” below). With the InfC system, on the other hand, the acoustic-phonetic information inside non-zero signal intervals for the *G* = 1 stimuli, as seen at the listener’s cochlear output, is the same as for the *G* = 0 corresponding stimuli [see property 3 of Section “Experiment II: infinite-clipping (InfC) of critical-band signals”], thus allowing a generalizing to tasks with higher perplexity.

With the PPC system Class-II stimuli, significantly more intelligible than the corresponding NoΘ stimuli, are the NoΘ + ChΘ stimuli. The existence of residual acoustic-phonetic information in the ChΘ signal [item 5 of Section “Experiment I: peak position coding (PPC) of critical-band envelopes”] implies that association between the functions of parsing and decoding still exists, thus preventing a decisive validation of the role of input rhythm in syllabic parsing *per se*. With the InfC system, on the other hand, the acoustic-phonetic information inside non-zero signal intervals, as seen in the listener’s cochlear output, remains the same for all values of *G*, e.g., for any prescribed degree of temporal envelope fluctuations [see property 3 of Section “Experiment II: infinite-clipping (InfC) of critical-band signals”], implying a clear disassociation between the roles of parsing and decoding.

### Perplexity of corpus

One drawback of using semantically plausible material (such as TIMIT or the Harvard-IEEE sentences) in perceptual studies that measure word error rate is the ability of listeners to guess some of the words using contextual information. Semantically unpredictable sentences (SUS) make it more difficult for the listener to decode individual words on the basis of semantic context. For this reason, Ghitza and Greenberg (2009) used SUS material in which the sentences conformed to standard rules of English grammar but were composed of word combinations that are semantically anomalous (e.g., “Where does the cost feel the low night?” and “The vast trade dealt the task”). The SUS is a high perplexity corpus.

In preliminary trials with the PPC system an attempt was made to use the SUS corpus. Informal listening revealed that, because of the extensive removal of acoustic-phonetic information in generating the NoΘ (see Section “The PPC System vs. the InfC System”), NoΘ + ChΘ stimuli became hardly intelligible. This finding led to the selection of a seven-digit string corpus for this study – a low perplexity corpus but without context. On the other hand, informal listening to SUS stimuli generated by the InfC system showed that for a range of *G* values the stimuli are intelligible. Extending Experiment II to SUS material is beyond the scope of this study.

### Interpretations

The behavioral data presented here show that intelligibility is improved by reinstating the input rhythm, either by adding a ChΘ (or a GlbΘ, to a lesser degree) signal to the NoΘ signal (Experiment I), or by reducing the Gain parameter from *G* = 1 to *G* = 0.5 or = 0.8 (Experiment II). What neuronal mechanism is capable of exploiting the re-inserted information in such an effective manner?

Our interpretation of the data argues for a crucial role of theta in syllabic parsing. We suggest that for stimuli with flat critical-band envelopes the tracking mechanism of the neuronal theta oscillator is deactivated. Consequently, the hierarchical window structure is situated in idle mode, i.e., not synchronized with the input, resulting in deterioration in performance. Reinstating the input-rhythm information (either by the PPC or by the InfC system) revives theta tracking hence the recovery of the synchronization between the window structure and the input, resulting in improved performance. This interpretation is consistent with the capability of the Tempo model to account for the behavioral data of Ghitza and Greenberg (2009), as detailed in Ghitza (2011).

Another possible interpretation of the data is in line with the classical view that assumes a direct role, *in the decoding process per se*, of neural onset responses to acoustic edges such as CV boundaries. According to this view phones are extracted from waveform segments (dyads) “centered” at markers triggered by acoustic edges. (It is worth recalling that a dyad is the acoustic reflection of the dynamic gesture of the articulators while moving from one phone to the next.) This view is supported by psychophysical reasoning, inferred from acoustics, on the importance of acoustically abrupt landmarks to speech perception (e.g., Stevens, 2002). Indeed, re-inserting the input rhythm with the PPC system produce signals with acoustic edges (NoΘ + ChΘ and NoΘ + GlbΘ; Figures 4F,G), which enable activation of neuronal circuitry sensitive to edges. Yet, two observations pose a challenge to the hypothesis that these edges are part of the decoding process: (i) Re-inserting GlbΘ signals, with pulses situated either at CV boundaries or at mid vowels, resulted in similar level of performance, and (ii) Reinstating temporal fluctuations by using the InfC system produce signals with a marginal presence of acoustic edges (see Figures 7D,E), yet with high intelligibility. These observations imply that the *precise* location of neural responses triggered by acoustic edges have little or no effect on intelligibility.

We suggest a different role to acoustic landmarks, in line with our interpretation of the data, in which they are part of the parsing (rather than decoding) process. We argue that neural responses triggered by acoustic landmarks serve as input to the mechanism that tracks the input syllabic rhythm. Note that this hypothesis includes all classes of acoustic landmarks (e.g., vocalic landmarks, glide landmarks, acoustically abrupt landmarks; Stevens, 2002). Given the prominence of vocalic speech segments in the presence of environmental noise, it may be that vocalic landmarks are more important than others in securing a reliable theta tracking. If so, a theta cycle is robustly aligned with [Vowel]–[Consonant-cluster]–[Vowel] syllables, and phones are decoded in temporal windows defined by the cycles of the beta, entrained to theta (Ghitza, 2011).

## Conclusion

Intelligibility (in terms of digit and string-error rates) of the last four digits in seven-digit sequences was measured as a function of judiciously manipulated changes in critical-bands envelope flatness, while attending to the disassociation between parsing and decoding. We found that the intelligibility of stimuli with flat critical-band envelopes is poor. The addition of extra information, restricted to the input syllabic rhythm, markedly improves intelligibility. We suggest that flattening the critical-band envelopes prevents the theta oscillator from tracking the speech pseudo-rhythm, hence disrupting the function of syllabic parsing. We argue that by reinstating the input-rhythm information the tracking capability of the theta oscillator is restored, hence the recovery of synchronization between the input and the hierarchical window structure, which governs the decoding process.

In conclusion, this study provides empirical support for the hypothesized role of theta in syllabic parsing. It provides a further support to the hypothesis that neuronal oscillations are important in processing and decoding spoken language. No hypothesis about internal physiological processes can be fully validated using only psychophysical methods. Nevertheless, the perceptual consequences of the acoustic manipulations used in this study suggest a potential role for neuronal oscillations in speech perception and establishes a behavioral context for future brain-imaging experiments using comparable speech material.

## Conflict of Interest Statement

The author declares that the research was conducted in the absence of any commercial or financial relationships that could be construed as a potential conflict of interest.

## Supplementary Material

The Supplementary Material for this article can be found online at http://www.frontiersin.org/Language_Sciences/10.3389/fpsyg.2012.00238/abstract
